# Early warning of moral responsibility deficiency in personalized education: a machine learning approach

**DOI:** 10.3389/fpsyg.2025.1605453

**Published:** 2026-01-15

**Authors:** Yue Li, Yao Wang

**Affiliations:** 1 School of Public Policy & Management (School of Emergency Management), China University of Mining and Technology, Xu Zhou, China; 2 School of Marxism, China University of Mining and Technology, Xu Zhou, China

**Keywords:** college students, individualized society, moral responsibility, monitoring and warning, random forests

## Abstract

Moral responsibility is not only an important component of the moral standard system, but also a significant indicator for measuring the moral consciousness and moral level of one person. Our contemporary society has the characteristics of differentiation and individuation. The individual society inevitably causes the impact to the university student thought, simultaneously also proposed the new challenge to the university moral education work, therefore, making it urgent to strengthen the cultivation of moral responsibility among modern college students. This paper empirically investigates the current situation of ethical responsibility of college students using self-administered questionnaires and individual interviews. The study found that the overall situation of college students’ moral responsibility is good, but there are some problems, such as the unclear understanding of morality, unstable moral emotions, insufficient moral ability, and non-standard moral behavior. There are more significant differences especially in the political appearance, grade, professional, academic performance, and number of times of participation in collective activities. So, it is necessary to pay attention to the cultivation of college students’ moral responsibility today. Based on this, a deep learning-based early warning model of college students’ moral responsibility is established to realize timely early warning of college students’ lack of moral responsibility.

## 1 Introduction

Responsibility is the moral lifeblood that sustains human evolution, drives the sustainable development of society, and forms the essential foundation for the progress of civilization (Meng and Ran, 2017). Morality is the eternal theme of human civilized life. The education of moral responsibility is both the unchanging educational theme of human society throughout the ages and the main spiritual core and key in moral education. Hegel (2010) states, “What makes morality moral is all in having the consciousness of knowing that one has fulfilled one’s obligations.” Similarly, Kant (1986, 2013) points out that the moral value of an act motivated by duty does not depend on the intention it seeks to fulfill, but on the norm it prescribes. Moral responsibility is “the necessity of behavior arising from respect for the law.” Luo (1993) states: “Moral responsibility is a morally conscious moral obligation, a moral mission, to others and to society.” Moral responsibility is essentially the inner recognition of external moral obligations. Moral responsibility is an obligation that people are actively aware of and has a component of conscience. Moral obligation and moral responsibility are two manifestations of the same moral “command” outside and within people. Ma (2013) pointed out that the cultivation of moral responsibility among contemporary college students is a comprehensive and systematic project that requires joint efforts from schools, families, and society. From the perspective of schools, moral responsibility education should be integrated into curriculum teaching, campus cultural construction, and moral education evaluation systems, fully utilizing new media to carry out moral responsibility education and emphasizing the exemplary role of teachers. From a family perspective, parents should understand the importance of moral responsibility, create a responsible family environment, and actively support college students in assuming moral responsibility. From a social perspective, the government should strengthen social moral governance, and the mass media and every citizen should fulfill their moral responsibilities in public life. In the education of college students, Romero et al. (2024) analysed the impact of these factors on academic performance during exams in a technical college in southwestern Spain, with a particular focus on environmental conditions, identified as a relevant and independent variable; Rivera et al. (2024) found that parents’ practices of engagement in students’ learning, discussions for students’ future, emotional support, students’ media consumption, disciplinary practice, and support for students’ autonomy vary in multifold ways as a function of their backgrounds; Suartama et al. (2024) found that in an era characterized by digitalization and the rapid development of technology, content, infrastructure, human, and environmental resources, it is essential to create an open and pervasive learning environment (PLE); Youssef and Alibraheim (2024) set out to investigate Saudi Arabian graduate students’ levels of anxiety related to statistics and their use of self-regulated learning strategies. Based on the correlation, it can be found that college students’ education is affected by all aspects of society.

Nowadays, Chinese society has the characteristics of differentiation and individualization (Yan and Zheng, 2012). In today’s individualized society, individuals have “detached” themselves from these traditional constraints, broken the standard patterns, and diverse lifestyles have emerged. Young people in an individualized society pay more attention to “self-actualization,” “personal happiness” and “quality of life.” When moral education is entirely collective-oriented and neglect the feelings and rights of individuals, it is easily regarded as a form of “preaching” and “moral coercion,” thereby leading to psychological rejection and rebellion. College students are increasingly finding that the moral norms they have learned cannot be applied to the social structure of their lives or future social structures. Individualized society inevitably has an impact on the thinking of college students, while also posing new challenges to moral education work in universities. Some college students lack awareness of moral responsibility, lack a clear attitude towards moral responsibility, and therefore cannot regulate their behavior according to moral standards (Xia and Zhou, 2019). Today’s society is a very intelligent society, deep learning technology has begun to be widely used in student education and achieved good results (Dai et al., 2020; Du Boulay, 2016; Cukurova et al., 2019; Du Boulay, 2019; Hughes, 2019; Kay and Kummerfeld, 2019), and has achieved fruitful results (Kitto and Knight, 2019; Luckin and Cukurova, 2019; Sellar and Gulson, 2021; Sharma et al., 2019; Wang and Wang, 2022; Webb et al., 2021; Zhang et al., 2023).

Therefore, considering the current social context, this paper examines the status and key influencing factors of moral responsibility among college students in an individualized society. It further establishes a predictive model based on the random forest algorithm to identify, in a timely manner, students who exhibit deficiencies in moral responsibility.

## 2 Theories related to individualized society and moral responsibility

### 2.1 Individualized theory

The so-called “individualized society” is inseparably related to Individualism and modernity in terms of its ideological sources. The German sociologist Tonnies and Lin, 2006 believed that one of the main tendencies of postmodern social life is individualization, which refers to a single individual intention. Realizing that one’s own personality, values and purposes can only develop by breaking free from the community that restrains them. In *The Individualized Society*, the British sociologist Bauman and Tester, 2001 simply referred to this social structure form as “the individualized society” and analyzed and pointed out that individualization carries the liberation of the individual. That is, to be liberated from the certainty of social attributes that belong to oneself, are acquired through genetics, and are inherent. This change is rightly regarded as the most obvious and potential feature of the modern situation (Bauman, 2002). In an individualized society, due to the acceleration of liberation, “de-traditionalization” is also accelerating. Individuals are gradually “detaching from the historically prescribed social forms and obligations in the traditional context of governance and support” (Ulrich and He, 2004). From the perspective of “re-embedding,” “individualization” means that the weight of life falls entirely on the individual. Life has become a completely personal and private matter. Each person relies on their individuality to anticipate and calculate the future course of life (Jin, 2004). In an individualized society, from the perspective of “de-personalization,” individuals “create their own lives by following the crowd” (Ulrich and He, 2004).

The individual is a concept full of questions and prone to causing oneself trouble. However, no matter what, it is still like other terms and is part of the words expressing our living conditions. Not to mention that no matter what explanation is related to the individual, it is bound to have an inseparable concentration with the connection between the individual and society, rather than just the individual himself. Bauman highly appreciated the analysis of the relationship between the individual and society by scholars such as Simmel, noting that their insights broke free from the rigid modern frameworks established by Hobbes regarding the individual–society dynamic (Bauman, 2002).

Individualization can thus be understood as the ongoing process through which individuals protect, realize, and continuously express their uniqueness. Moreover, the human experiences of birth, aging, illness, and death, as well as the stability and turbulence of society, and its independent yet interdependent structures, have always constituted a complex and evolving condition. Perhaps it is precisely because of the above state that throughout the course of human development, there has been a continuous process of acceptance and rejection throughout history, which has led to the logical transformation from a social-centered approach to an individual-centered one today. Just as discussing personal development is inseparable from society, the speed, scale and mode of the individualization process are always closely related to the degree of human understanding of nature and the functional differentiation of social structure (Bauman, 2002).

### 2.2 Moral responsibility in an individualized society

It is generally believed that the process of individualization has the following four major characteristics: (1) Eliminate the traditional model; (2) Separate individuals from and embed them within the system; (3) Pursue the popular idea of “living for oneself”; (4) Internal Manifestations of Difficulties within the System. From the current perspective, there are four distinct features of the individualization process that best highlight Chinese characteristics: (1) The situation breaks away from tradition and integrates with the collective; (2) The institutionalized withdrawal and re-integration of the current system are prominently reflected in the vigorous urbanization process. (3) Under the current circumstances, the personal ideology of “living for oneself” seriously highlights the typical melody of individualism; (4) In the current process of personalization, there are some risks within the system. Society and social trust are in a high-risk environment about to transform, and individuals are more sensitive and prone to anxiety symptoms (Yan and Zheng, 2012).

Under contemporary social conditions, the ongoing differentiation of society has resulted in a corresponding diversification of the moral system. Individuals in different occupational fields undertake varying forms of social responsibility, and public conceptions of moral responsibility have undergone significant transformation, exhibiting a complex and evolving developmental trajectory. Human beings are products of society, and so too are their moral responsibilities. Moral responsibility arises from the historical development of human society and serves as a defining feature that distinguishes humans from animals. From a historical perspective, morality—regardless of its specific form—is not determined by nature or by the will of individuals but is shaped by the social structures and cultural contexts in which it emerges. Likewise, the forms of responsibility embodied in contemporary morality depend fundamentally on the social environment of the present. All moral cultures are rooted in their unique historical conditions. Therefore, moral responsibility education must evolve in accordance with societal change to remain relevant and effective (Wang, 2019).

## 3 Questionnaire survey and result analysis of college students’ moral responsibility

This paper investigates the current situation of moral responsibility of college students in China at this stage in the form of a questionnaire on moral responsibility of college students. This investigation and research employed the strategy of stratified sampling. Scientific sampling surveys were conducted on students of all grades at China University of Mining and Technology, including those from different majors such as liberal arts, science, engineering and management, to ensure the scientific and reasonable nature of this research. The questionnaire on the current situation of college students’ moral responsibility has 40 questions, among which 1–30 questions are multiple-choice questions on moral cognition, moral emotion and moral ability, each question has five options, and we will assign scores from 1 to 5 in these options, 31–39 are multiple-choice questions on college students’ moral behaviors, and this part of the question only analyzes the results without scoring, and 40 questions are subjective short answer questions.

The compilation of the questionnaire on college students’ moral responsibility and the setting of the score standards mainly refer to the “Questionnaire on the Current Situation of Responsibility Awareness among College Students” written by Li (2007) from Shanxi Normal University and the “Survey on Students’ Responsibility Ethics Education” written by Su (2013) from East China Normal University.

### 3.1 Validity and reliability analysis of the questionnaire

To ensure the authenticity and reliability of the survey data and the validity of the survey results, it is necessary to verify the reliability and validity of the questions involved in the questionnaire. The main analysis results are as follows:

The questionnaire on the current Situation of College students’ Moral responsibility is a four-factor questionnaire with a total of 40 questions. The verification method adopted for the questionnaire in this study was the confirmatory factor analysis method, and the results obtained are shown in Table 1 below.

**Table 1:** Validity of credibility.

*x* ^2^	df	*x*^2^/df	NNFI	NFI	IFI	CFI	RMSEA
1440.45	485	2.97	0.93	0.92	0.91	0.92	0.072

As can be seen from the content shown in the table, all its indicators present an acceptable trend in the fitting model. Overall, the data and model fit well, confirming that the validity of the questionnaire structure on the current situation of moral responsibility among college students is within a reasonable range.

The reliability verification of the questionnaire in this paper adopts Cronbach’s Alpha coefficient test method. Generally speaking, a survey questionnaire can be considered reasonable only when the overall reliability coefficient of the questionnaire exceeds 0.8 and the reliability coefficient of the sub-questionnaires exceeds 0.7. Combined with the detection coefficient, the results are shown in Table 2.

**Table 2:** Analysis of credibility.

Name of the questionnaire	Dimension	Cronbach alpha
Questionnaire survey on the current situation of moral responsibility among college students	Total	0.88
	Moral cognition	0.78
	Moral sentiment	0.80
	Moral capacity	0.79

The table shows that the total reliability of the questionnaire is 0.88, indicating a good reliability index. The *α* coefficient value of the internal uniformity of standardization is also as high as 0.88, which overall indicates that the internal uniformity of the questionnaire is very good and the reliability of the questionnaire is reliable. In the questionnaire, the Cronbach’s Alpha coefficient obtained at the moral cognition level also reached 0.78. This data indicates that the internal consistency of the questionnaire is good. The coefficient at the emotional level is 0.8, and the coefficient at the moral ability level is 0.79. The data generally have reliable realistic reliability. Therefore, by integrating all the data, the author concludes that the reliability of this questionnaire has been confirmed to be reliable.

The rubrics and research results, as determined by the preliminary questionnaire, are shown below: If the total score of college students in the survey on the current situation of moral responsibility is above 100 (including 100 points), it is considered that college students have assumed their due moral responsibility. If the score is lower than 100 and higher than 75, it is considered that the university students do not fulfill their moral responsibility well, and there are deficiencies in their fulfillment of moral responsibility, which need to be improved. If the score is 75 or less, it means that the university students have serious problems in fulfilling their moral responsibility.

The standardized score delineated in the perceived moral responsibility section for college students is 32, and college students are considered morally responsible only if they score more than or exactly 32, and the opposite is morally responsible in a weak way. The standardized assessment score for the emotional component of college students’ moral responsibility is 35, and only those who score more than 35 (including 35) are considered to have a strong emotional component of college students’ moral responsibility, while those who score less than 35 are considered to have a weak emotional component of college students’ moral responsibility.

The standardized score for the moral responsibility component of the assessment for university students is set at 33, and only those who score more than or exactly 33 are considered to have a high moral responsibility, and the opposite is true for a low moral responsibility (see Table 3).

**Table 3:** General status of the current situation of moral responsibility of college students.

Score statistics for different situations	Min	Max	M	SD
	Statistic	Statistic	Statistic	Statistic
Total	42	143	98.25	22.15
Total score for moral cognition dimension	15	48	32.30	7.08
Total score for moral emotion dimension	13	47	35.10	7.32
Total score for moral ability level	14	48	32.98	7.16

By analyzing the questionnaire on the current situation of moral responsibility of college students, the highest score of this survey reaches 143 points, and the lowest score is only 42 points, which is a big difference, and the final result shows that 57.26% of college students have good moral responsibility education. Students who have received good moral responsibility education generally show a sense of voluntarily abiding by social morality, being honest and trustworthy, and having a strong sense of justice. They know how to respect others and be filial to their parents. Be willing to contribute in the collective, take the initiative to assume mistakes and fulfill promises, and internalize responsibility as a daily code of conduct. However, through the survey, we can also find that 36.51% of the college students have certain deficiencies in moral responsibility education; what is less optimistic is that 6.23% of the college students have serious problems in moral responsibility education, which needs our special attention.

### 3.2 Status of moral responsibilities of university students

#### 3.2.1 Current situation of moral cognition among university students

In terms of moral cognition, the scores among participating college students ranged from 15 to 48, indicating a 33-point difference, which reflects substantial variation in moral awareness across the sample. According to the previous criteria, those who scored 32 or above were considered to have strong cognitive ability, while those who scored the opposite were considered to have weak cognitive ability.

Data analysis shows that 57.8% of university students scored 32 or higher, demonstrating a relatively strong level of moral cognition, whereas 42.2% exhibited weaker moral understanding. Therefore, it is not difficult to find that the low level of moral cognition reaches as much as half of the population, and each university must pay extensive attention to it. In addition, it is worthwhile to recognize that many people with low moral cognition level are between 31 and 32 points, which is very close to the passing score of 32 points, so it seems that if the students themselves are a little bit disciplined, or receive external education, and form introspection, their passing requirements can be reached very soon. However, we can also see that not optimistic is that the level of moral cognition is seriously deficient in college students also exist, which must be moral educators and teachers in colleges and universities to increase educational efforts. Colleges and universities must formulate educational plans for the moral responsibility of college students, and need to be in the ideological aspect, to guide college students to establish the correct moral concepts.

#### 3.2.2 Moral emotion situation of university students at the present stage

In the questionnaire of moral emotion, the lowest score of college students is 13 and the highest score is 47, with a difference of 34 points. According to the classification criteria, 35 points and above indicate that college students have good moral emotion, on the other hand, those who scored less than 35 points are considered to have less good moral responsibility sentiments.

Through data analysis, it was found that 58.1% of college students demonstrated strong moral emotions, while 41.9% scored below 35 points. This indicates that nearly half of the students still exhibit weak moral responsibility emotions and require special attention. The data further show that many students with lower moral emotional scores fall between 33 and 34 points, which is very close to the qualification threshold of 35. With slight self-improvement or appropriate educational intervention, these students can reach the desired standard relatively quickly. Although only a small proportion of students scored below 25, enhancing the moral emotions of this group remains critical for strengthening moral responsibility education among college students.

#### 3.2.3 Current status of moral ability of university students

In terms of moral ability, the highest score is 48 and the lowest is 14, with a difference of 33 points. According to the above criteria, students with scores of 33 and above are morally ability, while those with scores of less than 33 have poor moral ability and have deficiencies in behavioral regulation.

This part of the data shows that the proportion of qualified students in the moral aspect is 56.4%; on the contrary, the proportion of students who have not reached the standard also reaches 43.6%. Through the survey data, many people with low moral cognition level are between 31 and 32 points, which is very close to the qualified 33 points, if the students themselves improve a little, and the teacher guides them a little, their qualified requirements can be reached soon. In addition, although students with scores lower than 25 are only a minority, educators must pay attention to the fact that the improvement of the moral ability of this part of college students is crucial to the education of college students’ moral responsibility.

#### 3.2.4 Status of moral behavior of university students

A questionnaire-based survey was conducted to assess the current state of moral behavior among college students. The analysis revealed that 50.9% of students demonstrated strong moral behavior, 39.7% exhibited moderate moral conduct, and 9.4% showed poor moral behavior.

Regarding the question, “Have you participated in the following activities during your college years?,” the results indicated that most students had engaged in moral or community-oriented activities to varying degrees. However, only a small proportion participated in multiple moral activities simultaneously, and a few had never taken part in any such activities. College students should actively and consciously participate in moral activities to make their due contribution to the society, especially in the poor areas. School teachers should cultivate college students’ awareness of participating in moral activities and enhance their enthusiasm to participate in moral activities, so as to make college students become qualified talents for the construction of socialist modernization.

The survey on the question “Do you often take the initiative to help your parents with household chores when you are at home?” The survey found that 19.3% of the students often help their parents, 30.5% will do what they can, 38.9% will not help unless their parents ask, and 11.3% never help. Filial piety and respect for parents is a traditional virtue of the Chinese nation, as an adult, college students should take the initiative to share the corresponding household chores for their parents and assume the corresponding family responsibilities. It can not only reduce the burden of parents, but also sharpen their hard-working spirit. For students who will not help unless their parents ask, this part of the students a little education, there will be a great improvement. For students who never help their parents, education needs to be focused on improving their ability to engage in moral behavior.

Through the survey on the question “If you put aside your own things to do something for the collective, what would you usually do?” The survey found that 16.5% of the college students were willing to do it, 41.8% thought I would do it if everyone else did it, 29.4% thought they would do it if it was beneficial to them, and 12.3% thought they would try not to do it if they could. When faced with the choice of doing something for the group at the expense of themselves, most of the students chose to go with the flow or to see if it could bring benefits to them. Doing things for the collective can be a good way to cultivate the spirit of teamwork. In today’s globalized society, competition is becoming increasingly fierce, and it is difficult to face and deal with complicated problems solely on one’s own, so we need to participate in more collective activities to cultivate teamwork and collectivism to adapt to today’s social development.

In today’s society, incidents of moral hesitation, often referred to as the “porcelain phenomenon,” occur frequently. In response to the question, “When you see someone in trouble, will you lend a helping hand?,” the survey revealed that 21.2% of college students would proactively offer assistance, 48.1% would decide based on the behavior of people around them, and 30.7% would refrain from helping out of fear of potential complications or misunderstandings. Faced with individuals in distress, many students rely on external cues rather than personal moral judgment. Therefore, universities should strengthen education and training in this area, enhancing students’ ability to discern genuine situations from deceptive ones. This will enable them to make accurate, rational moral judgments and translate ethical understanding into responsible action.

Through the survey on the question “What do you think is the relationship between personal ideals and social ideals?” The survey found that 31.8% of the college students think that they can achieve their ideal goals in realizing their social ideals, and 45.6% of them want to achieve their personal ideals first and then think about their social ideals, while 22.6% of them think that as long as they have accomplished their own ideals, their social ideals have nothing to do with me. Many college students lack a correct value, their purpose is often related to themselves, they cannot put the interests of the country and society in the first place, and they cannot make contribution to the construction of socialism. Schools should educate students to establish the correct values applicable to themselves, only a correct value can effectively plan their lives and guide them to make the right choices.

## 4 Influence of demographic variables on the current state of moral responsibility of university students

### 4.1 Influence of gender on the assumption of moral responsibility by university students

According to the analysis, gender differences were minimal; however, male students scored slightly higher than females across all three dimensions—moral cognition, moral emotion, and moral ability. There is still a slight difference between men and women in the analysis of the questionnaire on moral behavior, for example, through the question “What do you think is the relationship between personal ideals and social ideals?” The survey found that the realization of personal ideals in social ideals accounted for 31.8% of college students, of which 65.31% of college students were male.

### 4.2 Influence of political affiliation on college students’ assumption of moral responsibility

According to the statistical analysis of the questionnaire on the current situation of moral responsibility of college students, it is concluded that the average score of the party status college students is as high as 124.97, and the average score of the non-party status college students is only 96.84, with a Sig value of 0.0022. In terms of moral cognition the party status college students’ scores are 39.52, and the scores of the non-party status college students are 31.26, with a Sig value of 0.006; in terms of moral emotion, the average score of party status college students is 42.65, and the average score of non-party status college students is 34.01, with a Sig value of 0.0042; in terms of moral ability the average score of party status college students is 42.8, and the average score of non-party status college students is 31.57, with a Sig value of 0.0038. The specific data are shown in Table 4.

**Table 4:** Differences in political affiliation on the current status of moral responsibility of college students.

Variant	Total scores		Moral cognition scores		Moral emotion scores		Moral ability scores	
	Average value	Sig value	Average value	Sig value	Average value	Sig value	Average value	Sig value
Political party member	124.97	0.0022**	39.52	0.006**	42.65	0.0042**	42.8	0.0038**
Non-party member	96.84		31.26		34.01		31.57	

*p < 0.05; **p < 0.01; ***p < 0.001.

The analysis revealed significant variations based on political affiliation, affecting the overall moral responsibility score and all three dimensions—cognition, emotion, and ability. College students with party membership are significantly better than those without party membership in taking moral responsibility because they are more educated in all aspects of moral responsibility and have stronger self-discipline themselves.

### 4.3 The effect of grade differences on college students’ assumption of moral responsibility

According to the statistical analysis of the questionnaire on the current situation of moral responsibility of college students, it is concluded that the average score of senior (juniors and seniors) students is 110.73, and the average score of junior (freshmen and sophomores) students is 92.67, with a Sig value, 0.026. In the aspect of moral cognition senior college students have an average score of 35.39, and junior college students have an average score of 30.0, with a Sig value of 0.037; in terms of moral emotion, the average score of senior college students is 38.12, and the average score of junior college students is 32.85, with a Sig value of 0.023; in terms of moral ability, the average score of senior college students is 37.22, and the average score of junior college students is 29.82, with a Sig value of 0.018. The specific data are shown in Table 5.

**Table 5:** Differences in grade levels on the status of moral responsibility of college students.

Variant	Total scores		Moral cognition scores		Moral emotional scores		Moral ability scores	
	Average value	Sig value	Average value	Sig value	Average value	Sig value	Average value	Sig value
Senior	110.73	0.026*	35.39	0.037*	38.12	0.023	37.22	0.018*
Lower grades	92.67		30.0		32.85		29.82	

*p < 0.05; **p < 0.01; ***p < 0.001.

According to the results of the analysis, it can be seen that the grades differ in the overall level and in the three dimensions of moral responsibility, moral emotion and moral ability. College students in the higher grades (juniors and seniors) are better than those in the lower grades (freshmen and sophomores) in assuming moral responsibility because they have been in school for a longer period of time and have received more education on moral responsibility from school, have a more comprehensive understanding of what is happening in the country and society, and are themselves older and more mature mentally.

### 4.4 Influence of professional differences on college students’ assumption of moral responsibilities

According to the statistical analysis of the questionnaire on the current situation of moral responsibility of college students, it is concluded that the average score of liberal arts college students is 107.19, and that of science college students is 95.52, with a Sig value of 0.033. In terms of moral cognition, the average score of liberal arts college students is 34.75, and that of science college students is 30.55, with a Sig value of 0.042; in terms of moral emotion aspect, the average score of liberal arts college students is 37.03, and the average score of science universities is 33.72, with a Sig value of 0.027; in terms of moral ability, the average score of liberal arts college students is 35.41, and the average score of science college students is 31.25, with a Sig value of 0.039. The specific data are shown in Table 6.

**Table 6:** Differences in specialties in the current state of moral responsibility of college students SEAS science and engineering as academic subjects.

Variant	Total scores		Moral cognition scores		Moral emotion scores		Moral ability scores	
	Average value	Sig value	Average value	Sig value	Average value	Sig value	Average value	Sig value
liberal arts	107.19	0.033*	34.75	0.042*	37.03	0.027*	35.41	0.039*
SEAS	95.52		30.55		33.72		31.25	

*p < 0.05; **p < 0.01; ***p < 0.001.

According to the results of the analysis, it can be seen that the majors differ in the overall level and in the three dimensions of moral responsibility, moral emotion, and moral ability. Since liberal arts college students receive more education on moral responsibility than science and engineering college students, liberal arts colleges are slightly better than science and engineering colleges in assuming moral responsibility.

There are differences in the current status of moral responsibility of college students in the aspects of political affiliation, grade, and major. The current status of moral responsibility of party members, senior students, and students of arts and management is higher than that of other students in the four aspects of cognition, emotion, ability and behavior.

### 4.5 Influence of student achievement on college students’ assumption of moral responsibility

According to the statistical analysis of the questionnaire on the status of moral responsibility of college students, the average score of the students in the top 50% of the results was 112.68, and the average score of the students in the bottom 50% of the results was 106.90, with a Sig value of 0.023. In the aspect of moral cognition, the average score of the students in the top 50% of the results was 35.20, the average score of the students in the bottom 50% of the results was 32.51, with a Sig value of 0.033. Sig value is 0.033; in terms of moral emotion, the average score of students in the top 50% of the results is 39.79, and the average score of students in the bottom 50% of the results is 38.83, and the Sig value is 0.021; in terms of moral ability, the average score of students in the top 50% of the results is 37.69, and the average score of students in the bottom 50% of the results is 35.56, and the Sig value is 0.018. The specific data are shown in Table 7.

**Table 7:** Differences in specialties in the current state of moral responsibility of college students.

Variant	Total scores		Moral cognition scores		Moral emotion scores		Moral ability score	
	Average value	Sig value	Average value	Sig value	Average value	Sig value	Average value	Sig value
Liberal arts	112.68	0.023*	35.20	0.033*	39.79	0.021*	37.69	0.018*
SEAS	106.90		32.51		38.83		35.56	

*p < 0.05; **p < 0.01; ***p < 0.001.

According to the results of the analysis, it can be seen that the students’ grades differ in the overall level and in the three dimensions of moral responsibility, moral emotion, and moral ability. College students in the top 50 percent of students’ grades are slightly better than those in the bottom 50 percent of students’ grades in assuming moral responsibility because they have more self-control, etc., than those in the bottom 50 percent of students’ grades.

### 4.6 Influence of the number of participations in collective school activities on the assumption of moral responsibility by university students

According to the statistical analysis of the questionnaire on the current situation of moral responsibility of college students, it is concluded that the average score of students who participate in collective activities more than or equal to two times a month is 120.06, and the average score of students who participate in collective activities less than two times a month is 103.15, with a Sig value of 0.0033. In the aspect of moral cognition, the average score of college students who participate in collective activities more than or equal to two times a month is 38.78, the average score of In terms of moral emotion, the average score of university students who participated in collective activities more than or equal to twice a month was 41.31, and the average score of university students who participated in collective activities less than twice a month was 37.03, with a Sig value of 0.0051. In terms of moral ability, the average score of university students who participated in collective activities more than or equal to twice a month was 103.15, and the average score of university students who participated in collective activities more than or equal to twice a month was 103.15 with a Sig value of 0.0033. The average score of university students who participated in collective activities more than or equal to two times per month was 39.97, and the average score of university students who participated in collective activities less than two times per month was 35.10, with a Sig value of 0.0043. The specific data are shown in Table 8.

**Table 8:** Differences between professions in the current state of moral responsibility of college students.

Variant	Total scores		Moral cognition scores		Moral emotion scores		Moral ability scores	
	Average value	Sig value	Average value	Sig value	Average value	Sig value	Average value	Sig value
Group1	120.06	0.0033**	38.78	0.0063**	41.31	0.0051**	39.97	0.0043**
Group2	103.15		31.02		37.03		35.10	

*p < 0.05; **p < 0.01; ***p < 0.001.

Group1: Students who participate in group activities at least two times a month on average. Group2: Students participating in group activities less than twice a month on average.

According to the results of the analysis, it can be seen that the number of times of participation in school group activities varies in the overall level and in the three dimensions of moral responsibility, moral emotion, and moral ability. Since university students who have participated in school group activities more than or equal to two times have a stronger sense of teamwork than those who have participated in school group activities less than two times, those who have participated in school group activities more than or equal to two times are better than those who have participated in school group activities less than two times in terms of taking moral responsibility.

There are differences in the current status of moral responsibility of college students in these aspects of political affiliation, grade, major, academic performance, and the number of times they participate in school group activities. The moral responsibility status of students who are party members, senior students, students of arts and management, students with better grades, and students who participate in more school group activities are higher than other students in the four aspects of cognition, emotion, ability and behavior.

The identities of Party members and others are strengthened through organizational training to enhance their sense of social responsibility. Grade growth promotes mental maturity and moral judgment. Humanities and social sciences majors are more likely to inspire social ethical concern. Those who achieve excellent grades may adhere more strictly to norms due to moral self-discipline, but they may also fall into the trap of “refined self-interest.” Collective activities are the key at the practical level, directly cultivating the spirit of collaboration and a sense of collective honor, and transforming the perception of responsibility into action (Liu et al., 2011).

## 5 Exploration of the factors influencing the cultivation of moral responsibility among university students

University students—a group symbolizing vitality and potential—constitute the nation’s reservoir of high-quality human capital. As the builders of future thought and culture, college students are the outstanding successors who can carry the flag of the development of the motherland in the future. Therefore, college students’ awareness of moral responsibility is directly related to the development of society and the prosperity of the nation. Cultivating college students’ moral responsibility is influenced by many factors, including their own factors, family, school and social environment.

### 5.1 Deficiencies in self-cultivation

Today, we are all individuals, and there are many among us who have been differentiated without really being individuals enough to face the consequences of the differentiation process. As Ulrich Beck pungently points out in his book *The Society of Adventure*, for most of us the process of differentiation is actually “the piling up of contradictions and conflicts at the feet of the individual by experts, who kindly invite him or her to make critical judgments on the basis of his or her own perceptions of it all.” (Bauman, 2002).

Moral responsibility cognition is mainly the individual’s overall awareness of the norms of social moral responsibility and the initial understanding and realization that the self should take responsibility. “Self-awareness, as the individual’s awareness of himself and his relationship with society, is the regulator of individual thought and behavior, and plays an important role in the individual’s personality quality” (Zhang and Liu, 2016). The issue of moral responsibility of college students should be explored from the perspective of the self in the final analysis.

(1) Compared with mainstream social values, college students today demonstrate increasingly diverse and pluralistic value orientations. In the face of the current diversified society, some students blindly pursue material enjoyment and indulge in online games, they have no orientation to their own life, no career planning, and no lofty aspirations and lofty ideals. They attach importance to material enjoyment and despise spiritual pursuit, and generally show indifference to spiritual life. There is also a part of the students learning purpose utilitarian, heavy solicitation, light dedication, focus on personal interests and ignore the interests of others and the collective. The misalignment of values has directly led to the emergence of a crisis of moral beliefs among some students: they do not know how to be a human being, what kind of a human being they should be, they do not know how to plan their own lives, and they do not have a vision and outlook for the future.
(2) Compared with the consciousness of serving everyone, college students emphasize more on self-consciousness. Self-centeredness is a common problem among current college students, who think more about their own interests in interpersonal interactions, hoping that everyone is for me, but not for all. In the study, they are self-centered, only focus on the study to their own assessment of scholarships, research and other advantages, but do not care about the specific content of the study, but also ignored the traditional moral and cultural learning; in the dormitory, they hope that their roommates can clean up, and they will not take the initiative to undertake; in the class, they hope to get the relevant honors, and do not go to put themselves in the shoes of all of us; in the family In the family, they are not sharing the household chores for their family members.
(3) Compared with the expected sense of responsibility in society, college students’ sense of social responsibility is insufficient. At present, there is a common phenomenon among some college students: some students do not care about family life, are not enthusiastic about class affairs, do not pay attention to social problems, and are indifferent to the concept of collective honor and sense of social responsibility. Dormitory or public teaching area bathroom appears long flowing water, will pretend to turn a blind eye; the last to leave the classroom students can not do with the hands off the lights; in the library self-study, cell phones do not adjust the mute; see others littering will not stop in time… similar words and deeds are not uncommon in the campus.

### 5.2 Weakness of family guidance

The family is the first classroom of students, and parents are the first teachers of students; therefore, the influence of parents on students is subtle and will affect students through different forms and ways. Although family education is arbitrary, it is not unintentional. Parents’ values are limited due to the influence of different social classes. Parents are selective and conscious in imparting values to students.

(1) Influence of parents’ personal qualities. Parents must possess adequate educational literacy and moral awareness when guiding their children in developing a sense of moral responsibility. However, some parents lack sufficient knowledge, self-cultivation, or are negatively influenced by social environments, leading to deficiencies in their ability to provide effective moral guidance. This inadequacy can directly affect the quality and outcomes of students’ moral responsibility education. In addition, parents with lower educational attainment may inadvertently provoke resistance in their children, while those lacking proper parenting methods often adopt an authoritarian “know-it-all” stance, suppressing their children’s independent thought and imposing personal views upon them.
(2) Focusing on achievements and weakening rational and moral responsibility education. 00s youth group is the main member of colleges and universities nowadays, and these 00s students enjoy superior material conditions and parental pampering on the one hand and carry the high expectations of several generations of elders on the other. Some parents are devoted to satisfying their children’s needs and do everything for them, which makes them dependent, self-centered and weak-willed. At the same time, some parents do not fulfill their obligation to cultivate their children’s moral responsibility in education, and the mentality of expecting their children to be successful just makes them focus on test scores, and the phenomenon of “emphasizing intelligence over morality” is serious, resulting in some children’s concept of moral responsibility being vague, and their ability to distinguish right from wrong from good and evil being weak.
(3) Changes in family structure. In societies with low productivity and agricultural dominance, family members were closely connected through collective labor and shared responsibilities. However, as modernization and social mobility have accelerated, traditional patterns of family interdependence have weakened. Each family member now tends to pursue independent careers and personal goals, resulting in reduced mutual reliance and commitment within families. Moreover, in forming families, individuals increasingly prioritize personal emotions, interests, and benefits (Pan, 2023). During China’s ongoing process of individualization, family structures have diversified—including left-behind, migrant, divorced, and single-parent families—each presenting unique challenges to the cultivation of moral responsibility. These transformations underscore the need for adapted moral education approaches that address the evolving realities of family life.

### 5.3 Deviations in higher education

College students are at an important stage of forming and establishing a correct worldview, outlook on life and values, and the ideological and political theory courses in colleges and universities bear an important responsibility as the main channel for the education of college students’ moral responsibility. However, under the environment of individualized society, whether the ideological and political theory courses in colleges and universities can play their due roles has ushered in a new challenge (see Table 9).

**Table 9:** School approaches to fostering moral responsibility among university students (multiple choice).

School approach to cultivating moral responsibility in university students	Number of people
Thematic activities	202
Classroom teaching	481
Specialized lectures	186
Student Seminar	127
Travel on tour	52
Social practice	98
(sth. or sb) else	36

According to the current situation of the school pathway for the cultivation of college students’ moral responsibility, college students mainly rely on classroom teaching to obtain the education of college students’ moral responsibility, and the effect is relatively single.

(1) The gap between moral responsibility education and real social life. College students are deeply immersed in contemporary society, especially during this period of rapid social transformation. However, the outdated and overly static content of many ideological and political textbooks fails to keep pace with the evolving moral, cultural, and ideological realities faced by students. In general, the current materials used in colleges and universities remain overly theoretical, lacking relevance, engagement, and practical connection to students’ lived experiences. Consequently, the disconnect between classroom instruction and real-world moral challenges diminishes the effectiveness of moral responsibility education.
(2) Moral responsibility education in colleges and universities is a single way. At present, most of the ideological and political theory courses in colleges and universities are still taught in a kind of “bag of virtues” style, in which teachers instill the world’s widely praised virtues into students’ minds with a single way of teaching in class (Pan and Wang, 2018). Although in the current education process, social practice is more and more attention, but from the overall teaching environment, the proportion of social practice is still small, and did not form a complete system, moral education in colleges and universities can be said to remain in the “paper” stage, more single. This way of education ignores the students’ subjective initiative, and only lets the students passively accept the education. The arrival of individualized society, so that people pay more attention to independence than ever before, college students are more and more strong sense of self-generated subject. As a result, the moral responsibility education in colleges and universities ignores the concept of students’ subjectivity and gradually creates a contradiction between the ever-increasing subjective consciousness of college students. If this contradiction is not well resolved, it is easy to appear that college students are negative to the teacher’s preaching and the knowledge and action are not the same.
(3) The moral education role of teachers in colleges and universities is often undervalued. The team responsible for moral education in higher education institutions should encompass not only instructors of ideological and political courses, counselors, and class advisors but also teachers of various professional disciplines. Every educator, regardless of specialization, plays a vital role in fostering students’ moral responsibility. However, some professional course instructors narrowly view their duties as limited to academic teaching, assuming that moral education falls solely under the purview of Civics teachers and counselors. Moreover, a subset of teachers lacks firm moral convictions and cultural confidence, which can undermine the consistency and authenticity of moral guidance. In recent years, as more faculty members pursue study opportunities abroad, some have become influenced by Western ideologies, leading to a weakening of their own moral and cultural beliefs. Consequently, the values and messages they impart to students may become fragmented or biased, reducing the overall coherence and effectiveness of moral education in universities.

### 5.4 Complexity of the social environment

In addition to the factors of college students themselves, their families and colleges and universities, social factors also play an important role in the education of college students’ moral responsibility. Man is a social animal and can only survive and develop by relying on a certain group. For individuals, society is a cozy word, which can give individuals the meaning of life and make people’s short life leave long-lasting traces. But in the context of the beginning of social differentiation, “cultural life is no longer compensated by a collective conscience or a social reference unit, or, to put it more generally, by the fact that it is no longer the social class that takes the place of the identity group, or that the family, as a stable frame of reference, takes the place of the obligations of the social class. For sociality in social life, the individual himself becomes the unit of reproduction” (Yu, 2023). Society has begun to take on the characteristics of individualization: the emancipation of autonomy and self-consciousness, the shift from “living for others” to “living for oneself,” and the differentiation of social structures and job opportunities, which has led to increased mobility of the individual. In the survey, we also found that only 16.5% of university students would choose to work for the collective on their own initiative, believing that collectivism is higher than socialism.

(1) The influence of negative social customs. Since social existence shapes social consciousness, any study of college students’ moral responsibility must be grounded in the realities of contemporary social life. When asked, “Will you lend a helping hand when you see someone in trouble?,” 54% of the students chose “depending on the situation of the people around,” 27% chose “will take the initiative to lend a helping hand,” and 19% chose “will not, afraid of getting into trouble for being kind! The answer to this question is “No, I am afraid of getting into trouble for being kind.
(2) The new challenges of online media. The Internet as a global sharing open platform, everyone on the network has the right to speak, a variety of moral and cultural concepts co-exist. This has resulted in different moral evaluations of the same thing, which inevitably creates a conflict of moral culture. In response to the tiger-eating incident in Ningbo Zoo, Zhejiang Province, which occurred in January this year, two opposing voices appeared on the Internet: some people said that it was the responsibility of the zoo for its negligent management; some people said that the tourists who crossed into the park deserved to be punished for their crimes because he violated the rules in the first place; and some people said that the tiger that was killed was the most innocent one because it was only its nature to eat people, and it did not have any mistakes.

Traditional moral norms are unable to restrain some unmoral behaviors on the Internet because they are not adapted to the new environment of the Internet. In order to pursue economic interests and commercial profits, some people use the Internet to engage in unlawful behaviors such as violating other people’s privacy, spreading boring information, and engaging in information fraud, etc., which lose the bottom line of morality.

## 6 Warning of lack of moral responsibility of college students with multi- modal fusion in big data environment

### 6.1 Basic principles and steps of random forest algorithm

The decision tree algorithm serves as the fundamental base classifier within the random forest framework. Entropy represents the measure of uncertainty of random variables in decision tree system. In this paper, spanning tree algorithms such as ID3, C4.5, and C5.0 are chosen (Fang et al., 2011) to obtain entropy.

In the classification problem, assuming that there are a total of K categories in the classification results, the probability of appearing the k category is, then the expression of the Gini coefficient is $p_{k}$ as follows Equation 1 (Yin and Hu, 2014)


$$G(p)=\sum_{k=1}^{K}p_{k}(1-p_{k})=1-\sum_{k=1}^{K}P_{k}^{2}$$ (1)

For binary classification problem, this paper can also calculate the result more easily, assuming that the probability of the output belonging to the first class in the sample isp., then the expression of the Gini coefficient is as (Chen and Guestrin, 2016) follows Equation 2:


G(p)=2p(1−p) (2)

For a given sample D, suppose there are K categories, and the number of the k categories is *C*k, then the Gini coefficient expression for sample D is Equation 3:


$$\begin{array}{c} G(D)=1-\sum_{k=1}^{K}(\frac{|C_{k}|}{|D|})2 \end{array}$$ (3)

In particular, for sample D, if D is divided into two parts, D and D12, according to some value A of feature a, then the Gini coefficient expression of D under the condition of feature A is Equation 4(Wang et al., 2023):


$$\begin{array}{c} G(D,A)=\frac{D_{1}}{D}G(D_{1})+\frac{D_{2}}{D}G(D_{2}) \end{array}$$ (4)

However, a single decision tree is a weak classifier, often susceptible to overfitting, which in turn reduces the generalization performance of the model. Random forest algorithm is an extension of Bagging algorithm, and its base learner is decision tree model (Dai et al., 2022). To build a more diverse decision tree, randomness is introduced in the training process of random forest. The randomness of random forest is mainly divided into two kinds of randomness: the first kind of randomness is the randomness in the feature extraction process, and the second kind of randomness is the randomness of datasets election (Iqbal et al., 2022).

**Figure 1:**
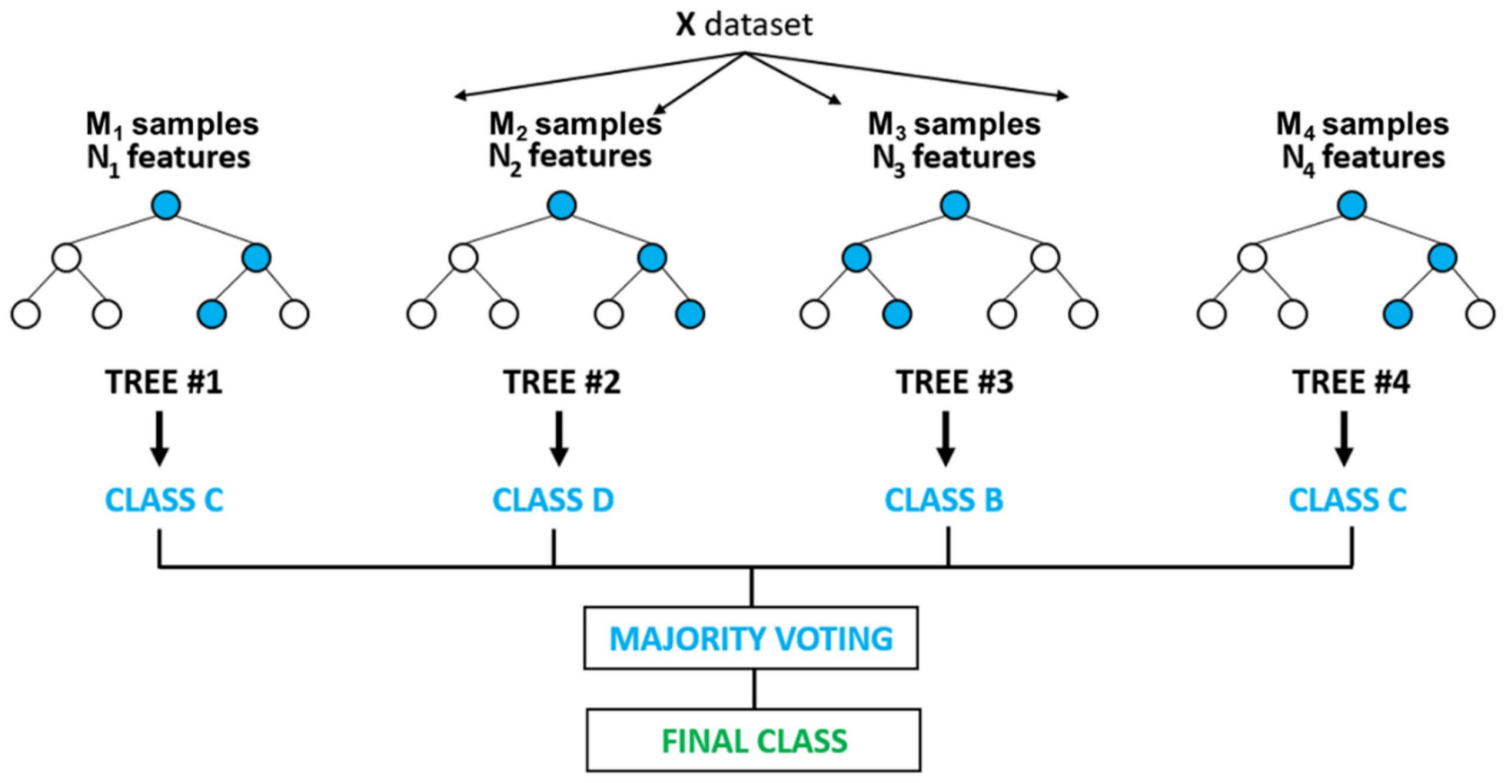
Workflow of random forest.

The prediction process of random forest is similar to that of decision tree in that it gets the final prediction result by starting from the root node, traversing each node in turn, and judging according to the eigenvalues of the nodes. In the classification problem, the random forest usually adopts the voting method, that is, the minority obeying the majority or taking the mode to make the final prediction (Zhang et al., 2022). The specific steps of random forest are as follows:

(1) Random sampling training decision tree: according to the comprehensive portrait, the four deduction items with the most deduction components are obtained as the four optimal features, and the corresponding samples of these four features are randomly selected.
(2) Adjust parameters to find the optimal features: add different numbers of new features to these four features and run the results.
(3) According to the relationship between the AUC of different parameters and the number of traits, the accuracy rate and the accuracy rate, select the characteristics and parameters with the best overall effect (see Figure 1).

### 6.2 Prediction and analysis of college students’ moral responsibility degree based on machine learning

#### 6.2.1 Data source and pre-processing

The data set used in this paper is the score of the questionnaire on moral responsibility of undergraduates in China University of Mining and Technology.

#### 6.2.2 Feature selection

**Figure 2:**
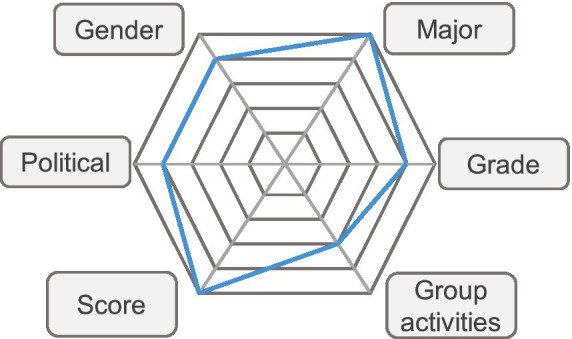
Predictive characteristics of students’ degree of moral responsibility.

In view of the large number of deduction items in the data set, it is easy to cause overfitting phenomenon. Therefore, it is very important to carry out effective feature selection. Based on the above analysis of influencing factors, this paper selects six characteristics, such as gender, major, grade, political status, achievement and the number of times of participating in school collective activities, as shown in Figure 2.

#### 6.2.3 Dividing training set and test set

**Figure 3:**
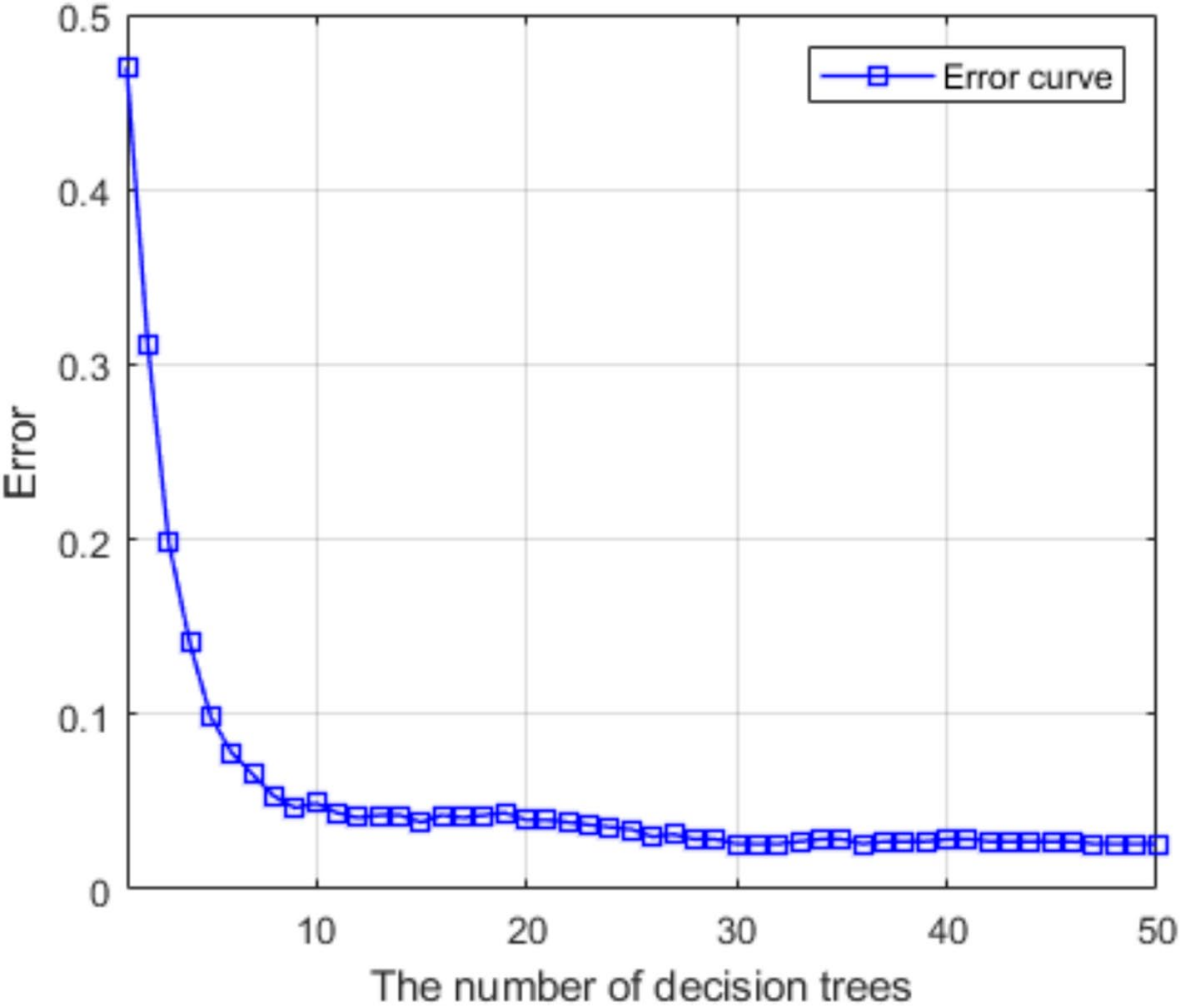
The error iterates with the decision tree.

Randomly select 90% of the data set to be divided into the training set and 10% of the remaining data set to be divided into the test set. The number of decision trees is 50, the minimum number of leaf nodes is 1, and the input feature dimension is 6. The iterative curve of the training error with the decision tree is shown in Figure 3. After 50 iterations of the decision tree, the error stably converges to 0.026. Furthermore, 10-fold cross-validation was adopted to demonstrate the generalization performance of the model. As shown in Table 10, the overall training error ranged from 0.017 to 0.042, with an average error of 0.0294, indicating a certain generalization performance of the model.

**Table 10:** Error distribution of 10-fold cross-validation.

Group number	Error	Group number	Error
1	0.026	6	0.035
2	0.042	7	0.031
3	0.023	8	0.027
4	0.039	9	0.030
5	0.017	10	0.024

#### 6.2.4 Evaluation indicators

The evaluation index selects three indexes: AUC, accuracy rate and accuracy rate. The value of AUC is equal to the area enclosed by the ROC curve and the *X*-axis, and the ROC curve is a curve with TPR as the vertical axis and FPR as the horizontal axis, describing the cost of obtaining a certain accuracy rate of the model. Where, TPR is the true case rate, that is, the proportion of samples with the true label of the sample as the positive example and the result as the positive example in the whole. FPR is the false positive case rate, that is, the proportion of samples whose true label is a negative example, and the result is also a positive example.

#### 6.2.5 Comparative research and result analysis

**Figure 4:**
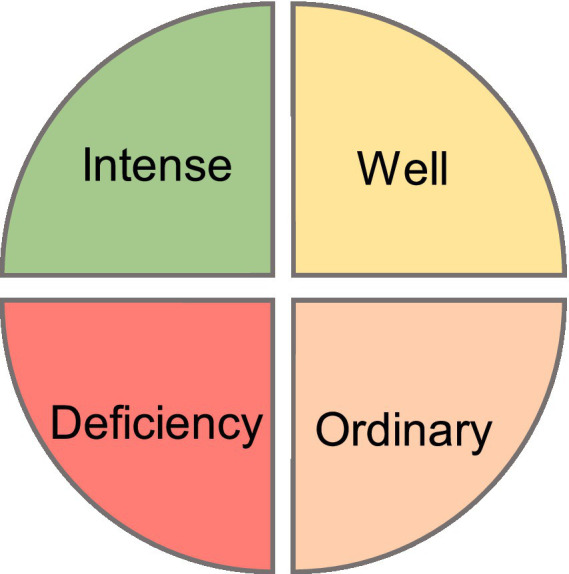
Lack of early warning of students’ moral responsibility.

Predict the degree of moral responsibility of college students in four different degrees: strong, good, average and lack, and give early warning to the lack of moral responsibility, as shown in Figure 4. After training the model of the random forest algorithm, five indexes such as AUC, accuracy rate, accuracy rate, recall rate and model training time were used to evaluate its prediction performance, and the prediction performance of XGBoost algorithm on this data set was compared. The training model of XGBoost algorithm still adopts the above method and selects the optimal feature number and features corresponding to XGBoost algorithm, as well as the optimal parameters. The results are shown in Table 11.

**Table 11:** Comparison of various indexes between random forest algorithm and XGBoost algorithm.

Different types of models	*R*	Accuracy	Average accuracy	Recall rate	Run time
Random forest algorithm	0.97	90.53%	91.16%	90.6%	1.198716 s
XGBoost algorithm	0.76	87.13%	95.30%	88.32%	9.19 s

The results show that AUC is used to measure the performance of the classifier. The closer the value is to 1, the better the performance of the classifier is. Both random Forest and XGBoost are excellent. Accuracy: The prediction accuracy of random forest algorithm is 90.53%, which is 3.4% higher than that of XGBoost algorithm; Accuracy: Random Forest is still higher than XGBoost algorithm. Recall rate: The ratio of the number of correct targets detected by the model to the number of actual targets, considering that it is better to predict a few wrong ones than to miss, to ensure that the characteristics of each user’s index are considered, from this dimension, the model with a high recall rate is completer and more accurate. Model running time: The running time of the random forest model is obviously much faster than that of XGBoost, indicating that the time complexity of the random forest is relatively small, indicating that the time complexity of the random forest algorithm is better than that of XGBoost. In summary, the prediction effect of the random forest is better than that of XGBoost algorithm. The reason why random forest performs better than XGBoost in recognition and classification is that random forest usually shows more robust performance for small samples, low dimensions, and noisy data, and can automatically process feature interactions. Force is more applicable to the mixed category characteristics such as gender and major involved in this study. The high accuracy of the random forest classification results means a reduction in misjudgment during student management, avoiding labeling normal students and protecting students’ mental health. A high recall rate means reduced underreporting and ensured that problem students can receive timely attention and assistance. A high AUC means clear classification and the ability to formulate differentiated educational plans for students of different levels.

### 6.3 Validation of the model

To evaluate the performance and effectiveness of the early warning and monitoring model for the degree of moral responsibility of college students proposed in this paper, simulation experiments are carried out. To ensure the representativeness of the samples, about 1,000 college students and their moral responsibility were randomly selected as the simulation data set.

**Figure 5:**
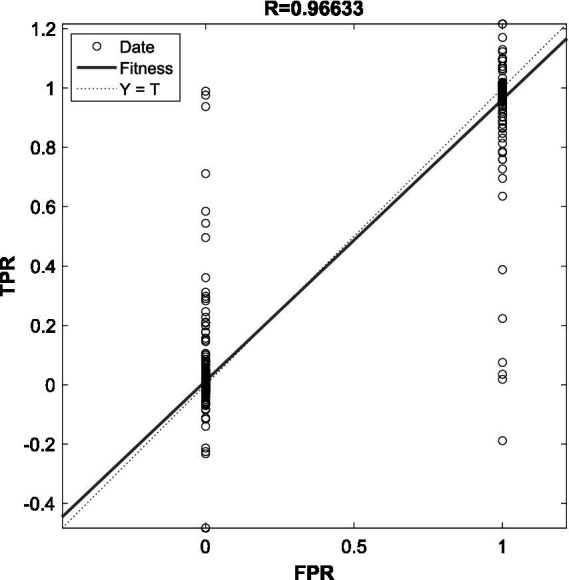
ROC curve of the simulation results.

**Figure 6:**
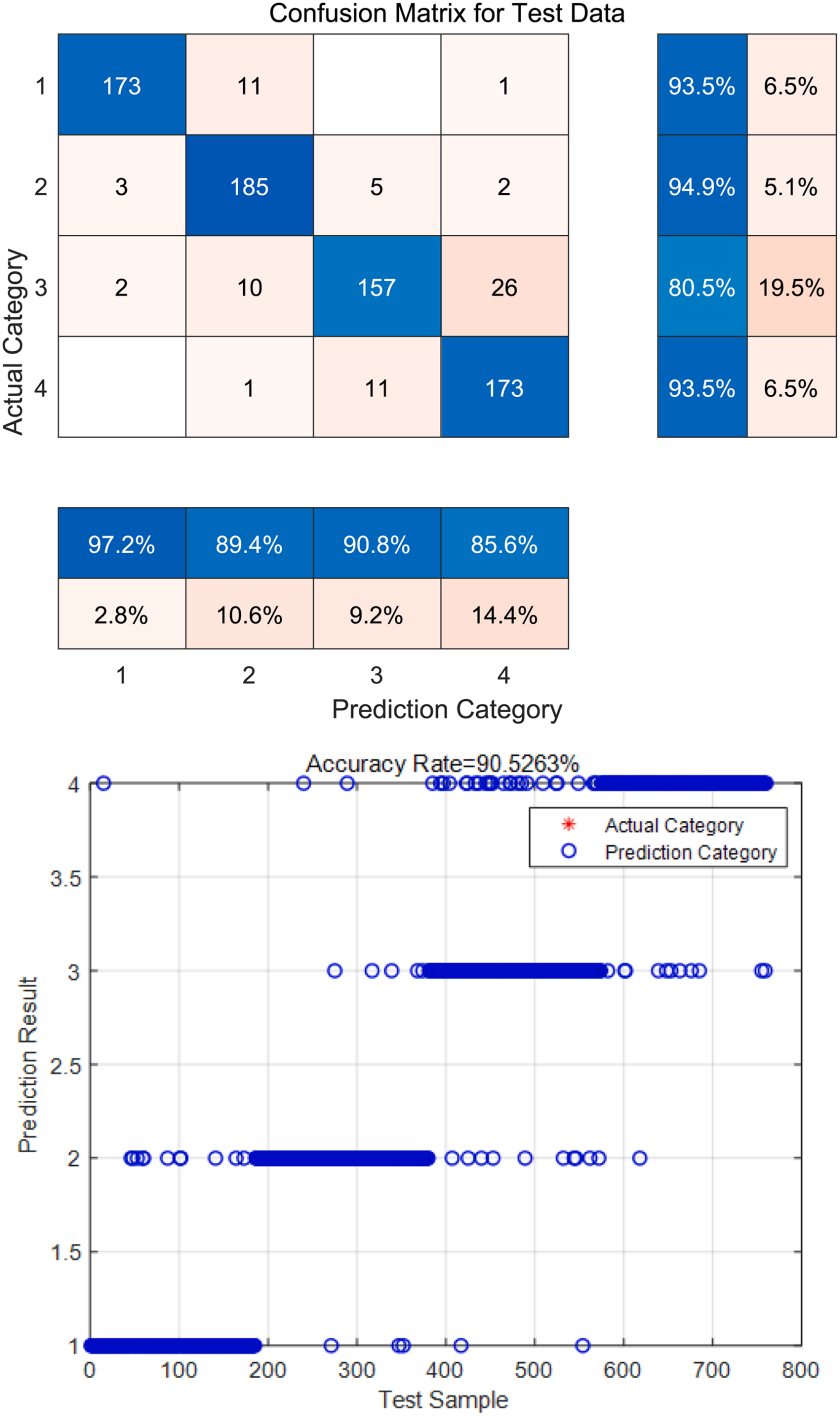
Confusion matrix of the simulation results.

In the simulation experiment, the early warning model of the random forest classifier is simulated. Through the prediction of the test set, the following simulation results are obtained: the accuracy rate is 90.53%. This indicates that the proposed model performs well in predicting the situation of moral responsibility of college students. In addition, the accuracy of the simulation data set is 91.16%, which shows that the model can accurately identify the different situations of moral responsibility of college students. In order to show the performance of the model more intuitively, the ROC curve was plotted and the confusion matrix was plotted according to the test set. The *R*-value of the ROC curve is 0.97, which proves that the built model has good classification ability. The model can correctly distinguish students with different levels of moral responsibility. That is, for any two students (one with strong moral responsibility and one with weak moral responsibility), the model has a 97% chance of correct identification. The results of the ROC index indicate that the system has high credibility, and schools can formulate educational intervention strategies based on the model results. The simulation results show that its recall rate is 90.6%, indicating that the model has a good ability to find those students who have real problems in moral responsibility of college students, indicating that the model can accurately and comprehensively estimate all the predicted information, and further verify the effectiveness of the model (see Figures 5, 6).

In summary, the simulation-based experimental results demonstrate that the proposed model exhibits high accuracy and strong predictive capability in assessing the moral responsibility of college students. The model’s performance on the simulated dataset was largely consistent with its theoretical expectations and even outperformed the test set results in terms of accuracy, AUC, and recall rate, indicating excellent robustness and reliability. Furthermore, the model—constructed upon the AHP-BP neural network comprehensive evaluation framework—provides a holistic representation of multi-attribute data, establishes a scientifically sound indicator system, and performs feature significance analysis combined with random forest classification of students’ moral responsibility indices. Through this process, optimal features and parameter configurations are identified, enabling efficient and precise prediction across large-scale datasets.

## 7 Discussion

The core limitation of applying machine learning to the research on the moral responsibility of college students lies in the data and model levels. In terms of data, self-reporting bias enables the model to learn answers that are expected to be “beautified” by society. Meanwhile, sample homogeneity and data from a single institution severely limit the universality of the conclusion, making it unable to represent student groups with diverse backgrounds. In terms of the model, the algorithm is good at identifying correlations rather than causal relationships, making it difficult to explain the intrinsic motivation of moral decisions. Moreover, its “black box” nature makes it hard to track the logical chain of responsibility judgments.

There may be some ethical issues when using machine learning for moral responsibility education. Applying machine learning to moral responsibility education involves multiple ethical risks. The primary issue is the solidification of values. Algorithms may replicate social biases in training data and package the designer’s values as universal standards, suppressing cultural diversity and critical thinking. Secondly, it may erode moral autonomy. Students perform “correct” behaviors to gain system recognition, turning morality into a scoring game and weakening their intrinsic drive and independent judgment ability. The last challenge is the difficulty in defining responsibility. When AI makes mistakes in providing moral advice, the attribution of responsibility becomes ambiguous, and educators may evade their duties of nurturing students. Technology simplifies moral complexity and transforms dynamic ethical exploration into static algorithmic output, which is contrary to the essence of education.

## 8 Conclusion

(1) The overall situation of college students’ moral responsibility is good, but there are some problems, such as the moral cognition is not clear, the moral emotion is not firm, the moral ability is insufficient, and the moral behavior is not standard. There are more significant differences especially in the political appearance, grade, professional, academic performance, and number of times of participation in collective activities. So, it is necessary to pay attention to the cultivation of college students’ moral responsibility today.
(2) A random forest algorithm-based early warning model of college students’ moral responsibility is established to realize timely early warning of college students’ lack of moral responsibility.
(3) To educators: The early warning model generated by the research can identify high-risk student groups. It is suggested that based on this, we shift from “flooding irrigation” to “precise drip irrigation” and carry out personalized intervention. For instance, micro-courses or case discussions on “individual initiative” can be pushed to students identified as having a tendency towards “dispersed responsibility.” For students who show a utilitarian tendency in moral decision-making, ethical workshops and in-depth counseling are provided to solve problems in their infancy.

To policymakers: It is suggested to abandon the single and static moral evaluation system and promote the establishment of a dynamic and multi-dimensional assessment framework for students’ moral development. By integrating the early warning model with campus data (such as academic performance, psychology, and activities), a comprehensive “digital portrait of student development” is constructed. Based on this, the allocation of ideological and political education resources is optimized, and more forward-looking and targeted campus support policies are formulated.

## Data Availability

The raw data supporting the conclusions of this article will be made available by the authors, without undue reservation.
